# Identifying features of risk periods for suicide attempts using document frequency and language use in electronic health records

**DOI:** 10.3389/fpsyt.2023.1217649

**Published:** 2023-12-11

**Authors:** Rina Dutta, George Gkotsis, Sumithra U. Velupillai, Johnny Downs, Angus Roberts, Robert Stewart, Matthew Hotopf

**Affiliations:** ^1^King’s College London, IoPPN, London, United Kingdom; ^2^South London and Maudsley NHS Foundation Trust, London, United Kingdom

**Keywords:** suicide, risk, electronic health records, language, assessment

## Abstract

**Background:**

Individualising mental healthcare at times when a patient is most at risk of suicide involves shifting research emphasis from static risk factors to those that may be modifiable with interventions. Currently, risk assessment is based on a range of extensively reported stable risk factors, but critical to dynamic suicide risk assessment is an understanding of each individual patient’s health trajectory over time. The use of electronic health records (EHRs) and analysis using machine learning has the potential to accelerate progress in developing early warning indicators.

**Setting:**

EHR data from the South London and Maudsley NHS Foundation Trust (SLaM) which provides secondary mental healthcare for 1.8 million people living in four South London boroughs.

**Objectives:**

To determine whether the time window proximal to a hospitalised suicide attempt can be discriminated from a distal period of lower risk by analysing the documentation and mental health clinical free text data from EHRs and (i) investigate whether the rate at which EHR documents are recorded per patient is associated with a suicide attempt; (ii) compare document-level word usage between documents proximal and distal to a suicide attempt; and (iii) compare n-gram frequency related to third-person pronoun use proximal and distal to a suicide attempt using machine learning.

**Methods:**

The Clinical Record Interactive Search (CRIS) system allowed access to de-identified information from the EHRs. CRIS has been linked with Hospital Episode Statistics (HES) data for Admitted Patient Care. We analysed document and event data for patients who had at some point between 1 April 2006 and 31 March 2013 been hospitalised with a HES ICD-10 code related to attempted suicide (X60–X84; Y10–Y34; Y87.0/Y87.2).

**Findings:**

*n* = 8,247 patients were identified to have made a hospitalised suicide attempt. Of these, *n* = 3,167 (39.8%) of patients had at least one document available in their EHR prior to their first suicide attempt. *N* = 1,424 (45.0%) of these patients had been “monitored” by mental healthcare services in the past 30 days. From 60 days prior to a first suicide attempt, there was a rapid increase in the monitoring level (document recording of the past 30 days) increasing from 35.1 to 45.0%. Documents containing words related to prescribed medications/drugs/overdose/poisoning/addiction had the highest odds of being a risk indicator used proximal to a suicide attempt (OR 1.88; precision 0.91 and recall 0.93), and documents with words citing a care plan were associated with the lowest risk for a suicide attempt (OR 0.22; precision 1.00 and recall 1.00). Function words, word sequence, and pronouns were most common in all three representations (uni-, bi-, and tri-gram).

**Conclusion:**

EHR documentation frequency and language use can be used to distinguish periods distal from and proximal to a suicide attempt. However, in our study 55.0% of patients with documentation, prior to their first suicide attempt, did not have a record in the preceding 30 days, meaning that there are a high number who are not seen by services at their most vulnerable point.

## Introduction

### Background

Individualising psychiatric care at times when patients are most at risk of suicide involves shifting research emphasis from static risk factors to those that may be modifiable with interventions (1, 2).

### Suicide risk assessment

Currently, risk assessment is based on a range of extensively reported risk factors gleaned from case–control studies using a psychological autopsy approach or nested within large register-based cohort studies (3). Critical to dynamic suicide risk assessment is an understanding of each individual patient’s health trajectory over time.

### Electronic health records

Medical records provide a chronological account of healthcare and are designed to be updated by all members of the multidisciplinary team (4). With the adoption of Electronic Health Records (EHRs) in both outpatient and hospital-based care by many healthcare providers, there is an opportunity to generate artificial intelligence-based insights from the analysis of the entire patient record (5). There are of course potential challenges posed owing to the accuracy of data held, consistency of recording, and comprehensiveness of data completion (6). However, for clinicians, it can also be the metadata which is revealing. For example, little is reported about how EHR documentation changes prior to a suicide attempt or even the proportion of those known to services who have a recorded interaction in the time preceding a suicide attempt (7).

### Data-driven modelling

Recent studies using longitudinal EHRs to predict suicidal behaviour have moved away from traditional statistical analyses (which typically produce an algorithm of up to 20 factors (8) but often overfit to high-dimensional data). The move has been towards data-driven modelling approaches, such as the Naïve Bayesian classifier model (9), Random forests (10, 11) or ensemble learning, including combination predictions from elastic net penalised logistic regression, Random forests, gradient boosting, and neural networks (12).

### Natural language processing

Other approaches have analysed the text used in EHRs using natural language processing (NLP) to investigate whether it adds predictive value to existing suicide risk models, e.g., extracting clinical concepts that are then annotated with Concept Unique Identifiers (CUIs) from the Unified Medical Language System (UMLS) (13) or using a general-domain sentiment analysis tool to assess the utility of words conveying positive or negative emotion (i.e., valence) (14). To make the unstructured text computable, existing standard vocabularies [e.g., those used in healthcare and biomedical sciences for UMLS (13)] or curated lists of subjectively valence-conveying terms (e.g., an included lexicon of nearly 3,000 words annotated for polarity [negativity vs. positivity rated −1 to +1 (14)] are used.

### Scientific approaches in this study

We investigated whether the rate at which EHR documents are recorded per patient is associated with a suicide attempt. We hypothesised that by aligning to the first suicide attempt, it would be possible to identify an increasing trend in EHR documentation detecting the impending occurrence of a suicide attempt.

We realised one avenue that had not been explored in the field was domain experts themselves creating the categories based on available text to investigate whether there are differences in word usage between times proximal and distal to a suicide attempt.

As a complementary analysis to this “presence/absence” method, where the more local context around the word usage is, by definition, lost, and where very common words such as prepositions would not be captured, we also performed what we call an “*n*-gram frequency analysis.” Changes in the length and frequency of sequential co-occurrence of words (n-grams) have been studied for other clinical use cases in the unstructured content of EHRs, e.g., oncology notes (15). We hypothesised that n-grams related to third-person pronoun use would emerge with increasing frequency nearer the date of attempted suicide as had been found in the clinical notes of veteran outpatients who died from suicide, compared to those who did not (16).

To overcome the challenges inherent to the way the data are locked in the free text of EHRs, we report on three measures to compare the proximal and distal periods from a suicide attempt: (i) rate of EHR documentation, (ii) categorisation of words used by clinicians in free text, and (iii) n-gram frequency related to third-person pronoun use.

## Materials and methods

We studied mental health service utilisation data and clinical free text data from 30 days time windows prior to suicide attempts and compared these to distal periods of lower risk. The selection of a 30 days window was based both on clinical knowledge of changes in mental health prior to an attempt and because 30 days windows have been used in other studies to train predictive models of suicide attempt risk (17).

The cohort of patients assessed in this study was assimilated from the South London and Maudsley NHS Foundation Trust (SLaM) Biomedical Research Centre (BRC) Clinical Record Interactive Search database: a case register system that provides de-identified information from electronic health records (EHRs) relating to secondary and tertiary mental healthcare services across 4 boroughs of South-East London and over 50 specialist services (18). SLaM provides secondary mental healthcare to a population of approximately 1·8 million residents of Lambeth, Southwark, Lewisham, and Croydon and national specialist services. EHRs have been used comprehensively across all SLaM services since 2006. CRIS was established in 2008 to allow searching and retrieval of full but de-identified clinical information for research purposes with permission for secondary data analysis, approved by the Oxfordshire Research Ethics Committee C (reference 08/H0606/71 + 5). As of 10 February 2017, CRIS contained clinical records on 277,700 patients, 176,242 of whom had contact with SLaM between April 1, 2006 and March 31, 2013, the period of interest for this study, for which there were data available, with at least one documented “event”, or attachment, e.g., correspondence, in common word processed format. The event field of the EHR is used by clinicians to enter notes regarding a patient’s history, mental state examination, progress, or risk in free text format.

CRIS has been linked with Hospital Episode Statistics (HES) data for Admitted Patient Care. HES is a national administrative database containing patient-level records of all admissions to NHS hospitals in England. Static extracts of HES data are linked to CRIS data within the Health and Social Care Information Centre and provided to the SLaM BRC with all identifiers removed. HES data are available within CRIS for all patients who have had any contact with SLaM services since 2006, regardless of where they were living at the time of their hospital use. Linked HES data were available up to 31 March 2013. Each record in HES corresponds to a finished consultant episode, during which a patient is under the care of an individual consultant. A hospital admission comprises a continuous time period of HES episodes.

### Identifying hospitalised suicide attempts

**Figure 1:**
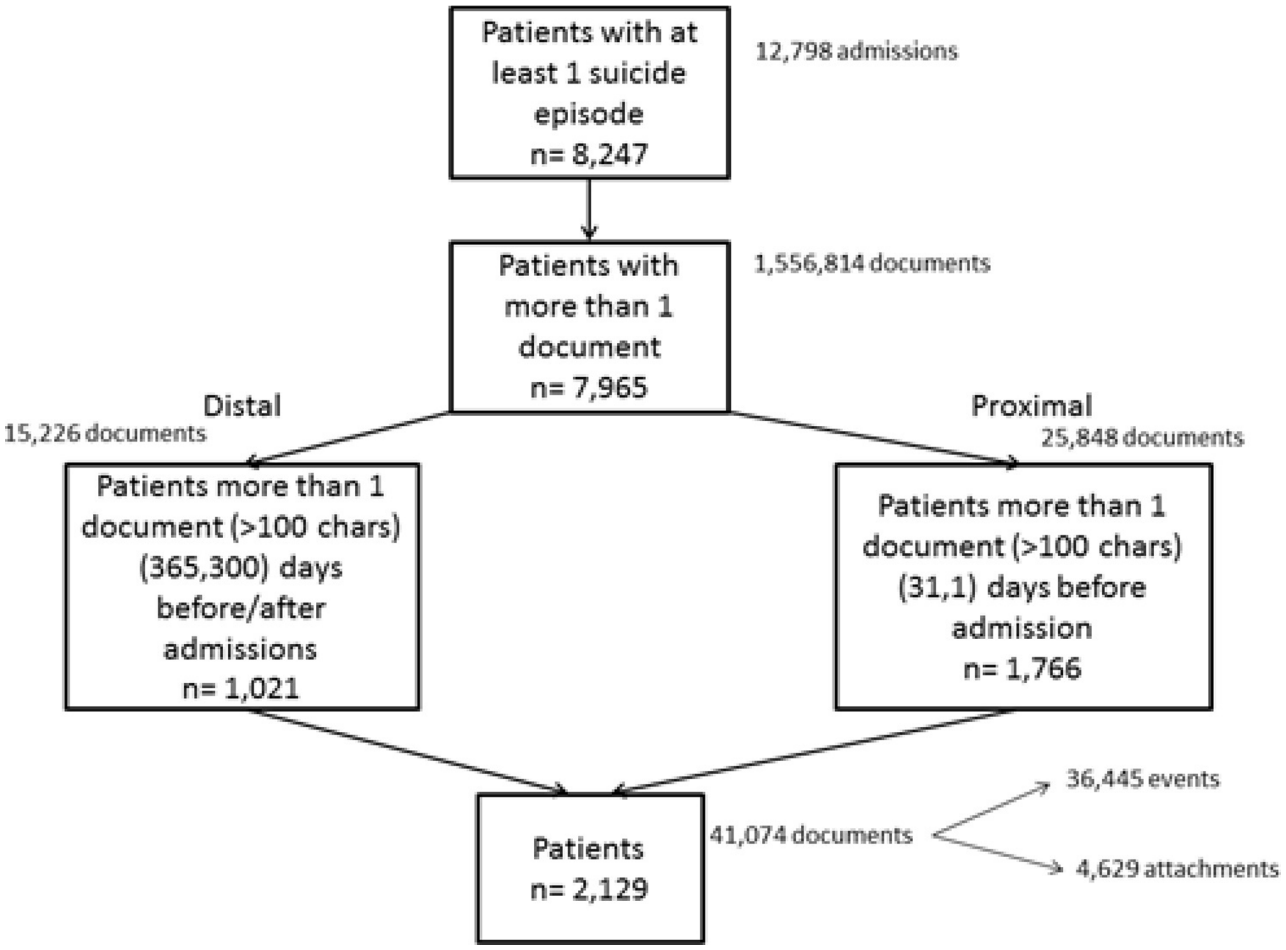
Derivation of the patient cohort and corpora of distal and proximal documents.

Our study included event and attachment data from *n* = 8,247 SLaM patients who had at some point between April 1, 2006 and March 31, 2013 been hospitalised with a HES ICD-10 code related to attempted suicide (X60–X84; Y10–Y34; Y87.0 / Y87.2; as described in http://www.ons.gov.uk/ons/dcp171778_351100.pdf). For these patients, all HES admission data were retrieved, even if they were unrelated to suicide. Some episodes formed part of a suicide-related admission or a completely different, non-suicide-related admission. Episodes were consolidated into hospital spells covering a patient’s total length of stay in a hospital (i.e., a hospital admission) and from these only suicide-related admissions (*n* = 12,798) were retained for analysis (see Figure 1). We included 7,965 patients with at least one event or attachment available, about whom more than 1.5 million documents had been written.

### Rate of EHR documentation/monitoring level

We investigated whether the rate at which EHR documents are produced per patient is associated with a suicide attempt. For this analysis, we only considered the first HES-identified suicide attempt between April 1, 2006 and March 31, 2013 and aligned all patients by this date.

For any given date prior to the first suicide attempt, we defined monitoring level as the number of documents produced for each patient for a fixed time window. We also normalised this value by dividing it by the size of the time window considered. For example, in our approach, we considered a time window of 30 days; therefore, if 30 documents had been produced in the preceding 30 days, the average daily rate—denoted as *MonitoringLevel_30_*—equalled 1. We only considered each patient as under monitoring at a given date if there was at least one document prior to that given date.

### Proximal and distal corpora selection and pre-processing

**Figure 2:**
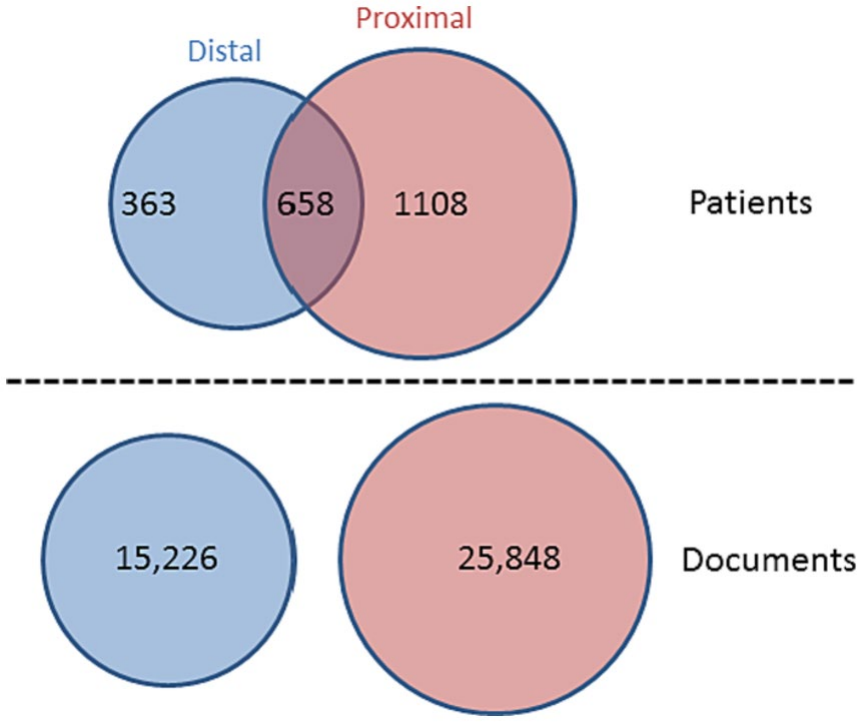
Venn diagram showing *n* = 2,787 patients contributing data to the analysis, with *n* = 15,226 documents pertaining to the distal period (between 365 and 300 days prior/following a suicide-related hospital admission) and *n* = 25,848 documents relating to the proximal period (between 31 days and 1 day prior to a suicide-related admission).

We aimed to compare documents entered by clinicians in two distinct time periods: (i) the *proximal* period comprising documents produced between 31 days and 1 day prior to a hospital admission linked to a suicide attempt and (ii) the *distal* period, including all documents created between 365 and 300 days prior to the first admission and all documents created between 365 and 300 days before any other admission, but *not less than* 300 days following their previous suicide-related admission (see Figure 2). For the proximal period, we retrieved 25,848 documents from 1,766 patients and for the distal period, we extracted 15,226 documents relating to 1,021 patients. 658 patients contributed documents to both the distal and proximal periods (see Figure 2). As we aimed to analyse the text in these documents, we only retained those documents that contained more than 100 characters.

### Word extraction for categorisation

We used standard corpus techniques to find the most discriminating words in the documents (19). We extracted the text from all 25,848 proximal documents and 15,226 distal documents. We applied Part-of-Speech (POS) tagging using spaCy^1^ and replaced all words with their POS label except for the words identified as nouns, pronouns, or verbs. We applied lemmatisation to the words that were retained. We examined each word to see if their presence (or absence) yields any discriminative power. For instance, if the word “overdose” is more prominent in the proximal period, we expect that documents that used this word at least once would be present more frequently in the proximal period. To assess words for their discriminability, we considered odds ratios, where objects are documents and their class is the period from which they originated.

We examined all words retrieved from our corpus and retained those words (*n* = 631) that had *p*-value ≤ 0.05 and odds ratio either lower than 0.66 or higher than 1.50. A senior clinician in mental health (RD) went through the list, excluding abbreviations (e.g., tc and pas), mentions of dates (e.g., 8th and 26th), times (e.g., 11 am and 5 pm), service-specific locations (e.g., Southwark and Ladywell), words of ambiguous meaning when not in context (e.g., paper—could be Mental Health Act (1983) Section paper, or paper used in Occupational Therapy activity; clean, clear—multiple meanings depending on context). *N* = 390 words were retained for human “topic modelling”.

We considered using computational topic modelling (20), but noted that computationally derived topics and representative terms are not always the same as the concepts used by clinicians (21). We therefore used a human-based topic model, in which clinician input was used to filter words and derive topics from those with discriminative power. We restricted the words considered for modelling to nouns and verbs as these are more likely to make a semantic contribution to the text. We also manually filtered out discriminative words that did not contribute to clinical interpretation.

RD curated the initial list by manually grouping them into clusters of similar meaning. RD formulated structural descriptions of each category based on empirical observations of the data (see Appendix 1). A second senior clinician (JD) was then given the precompiled list of categories and asked to assign all *n* = 390 words to them, without introducing any additional categories. The odds ratios for each group were then calculated as we had done previously for individual words. We considered a document as exposed if it contained at least one word from a given group.

### N-gram frequency analysis

We applied a machine-learning classification algorithm to the corpus, to classify each document as either distal or proximal (binary classification) and extracted the most informative *n*-gram features as found by the classifier.

An *n*-gram is a sequence of *n*-words in a text. For instance, for the word sequence, “*the patient is not suicidal.”* a uni-, bi- and tri-gram (1, 2, and 3) representation would be [“*the,”* “*patient,”* “*is,”* “*not,”* “*suicidal,”* “*.”*], [“*the patient,”* “*patient is,”* “*is not*,” “*not suicidal,”* “*suicidal*.”], and [“*the patient is*,” “*patient is not,”* “*is not suicidal,”* “*not suicidal.”*], respectively. This is a common model for representing text content in NLP classification tasks (22). We lemmatized the corpora using SpaCy and then applied the Naïve Bayes classification algorithm as implemented in the Python scikit-learn toolkit (23) using each of the three representations and then extracted the top 30 most informative features from each classification model. Informative features were those that contributed the most to discerning whether a document is distal or proximal.

The *n* = 90 resultant uni-, bi-, and tri-grams were then analysed and sorted with respect to their ORs in relation to their mean frequency of occurrence per document in the entire corpus. In this way, we were also able to analyse each feature with respect to whether it was informative for discerning a document as distal or proximal.

We analysed the n-grams in the following way: (i) an overall analysis of word types (part-of-speech and content), (ii) an analysis with respect to the feature’s OR, and (iii) an analysis with respect to *n*-gram content, e.g., whether or not similar words/word sequences were consistently scored as informative in the three representations.

## Results

Of the 8,247 SLaM patients who had at some time between April 1, 2006 and March 31, 2013 been hospitalised with a HES ICD-10 code related to attempted suicide (X60–X84; Y10–Y34; Y87.0/Y87.2), *n* = 4,607 (55.9%) were female, and the median age at first admission was 33 years (IQR 22–44; mean: 34.6 years and SD: 15.4 years).

### Documentation level prior to the first suicide attempt

Only 3,167 (39.8%) of patients who had made a suicide attempt had at least one document available in their EHR prior to their first suicide attempt. *N* = 1,424 (45.0%) of these patients had been monitored by mental healthcare services in the past 30 days. Yet the majority (*n* = 1,743; 55.0%) of patients with documentation prior to their first suicide attempt did not have an EHR in the preceding 30 days.

**Figure 3:**
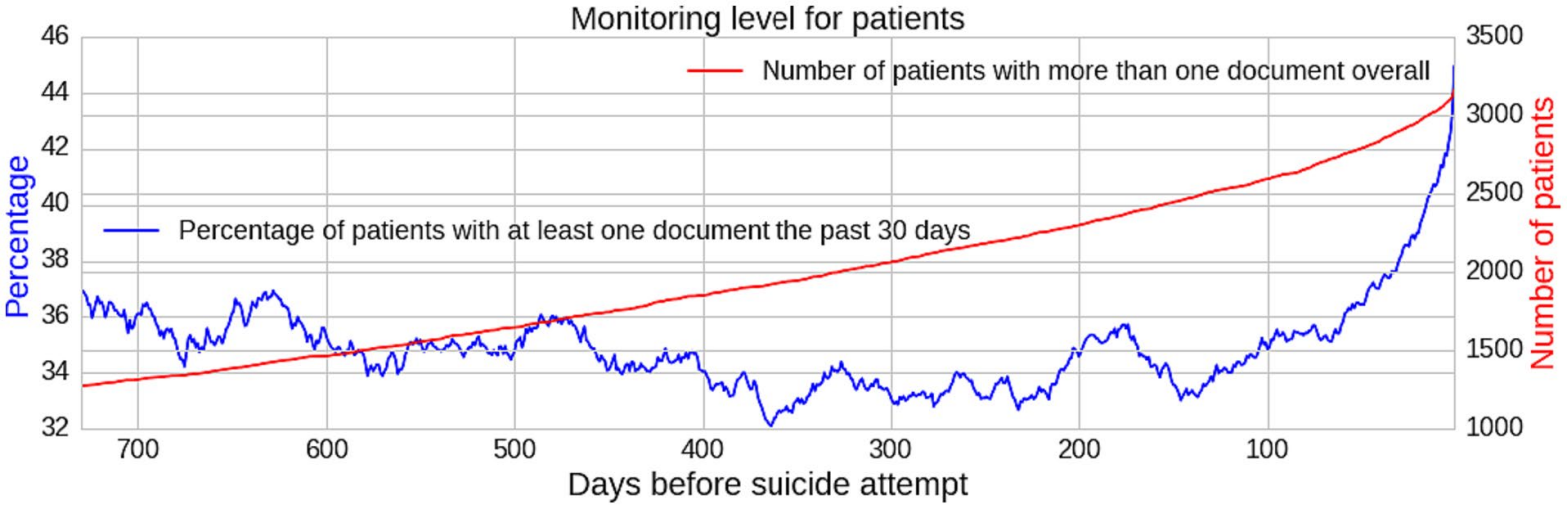
Graph showing monitoring level for patients indicating an increase in monitoring level proximal to a suicide attempt. Monitoring level numerator in blue and denominator in red.

The percentage of patients with more than one document in the preceding 30 days is generally within the range of 32.1–36.9%. However, from 60 days prior to a first suicide attempt, there is an exponential-like increase in the monitoring level in the past 30 days (increasing from 35.1 to 45.0%) (Figure 3).

### Comparison of document-level word categorisation between proximal and distal data

The list of *n* = 390 words retained for topic modelling was categorised into 17 groups (7 “protective” [PROT-A to PROT-G] for suicide attempt with OR < 0.66 (no. of exposed docs = 9,801); 10 “risk-related” [RISK-H to RISK-Q] with OR > 1.50 (no. of exposed docs = 62,118). (Refer to the Appendix 1 for comprehensive descriptions of each category, the number of words in each category, and the numbers of documents analysed with examples of words used in the EHR free text. The complete list of *n* = 390 words may be obtained from the authors upon request).

The groups vary in size: the smallest group containing *n* = 3 words (senior healthcare professional roles) and the largest *n* = 91 words (suicide “risk” terms and formal clinical distancing language). The odds ratios for each group were calculated, and these are summarised, along with precision, recall, and F1 scores, in Table 1.

**Table 1:** 17 categories of words used by clinicians in free text with ORs of proximal to distal use, with precision, recall, and F1 scores (for fuller descriptions of categories refer to Appendix 1).

Group	Category description	Odds ratio	Precision	Recall	F1 score
PROT-A	Care plan	0.22	1.00	1.00	1.00
PROT-B	Senior healthcare professional role	0.32	0.60	1.00	0.75
PROT-C	Chronic physical comorbidity / symptom	0.50	0.67	0.44	0.53
PROT-D	Treatment for drug addiction or depot treatment	0.55	1.00	0.80	0.89
PROT-E	Food/meals/activities	0.57	0.95	0.88	0.91
PROT-F	Positive connotations	0.58	0.56	0.75	0.64
PROT-G	Items used on ward	0.58	0.80	0.89	0.84
RISK-H	Items of clothing	1.54	1.00	0.89	0.94
RISK-I	Subheadings of clerking/diagnosis/psychiatric symptoms	1.62	0.77	0.80	0.79
RISK-J	Interventions	1.62	0.75	0.62	0.68
RISK-K	Time- or life event- or person/relationship-related	1.63	0.89	0.93	0.91
RISK-L	Suicide “risk” terms and formal clinical distancing language	1.64	0.79	0.79	0.79
RISK-M	Implement/mechanism of self-harm or suicide attempt	1.72	0.79	0.93	0.86
RISK-N	Negative connotations/judgemental language	1.72	0.86	0.63	0.73
RISK-O	Physical symptom or sign	1.84	0.60	0.67	0.63
RISK-P	Junior or multidisciplinary healthcare professional role	1.85	1.00	0.78	0.88
RISK-Q	Prescribed medications/drugs/overdose/poisoning/addiction	1.88	0.91	0.93	0.92

**Figure 4:**
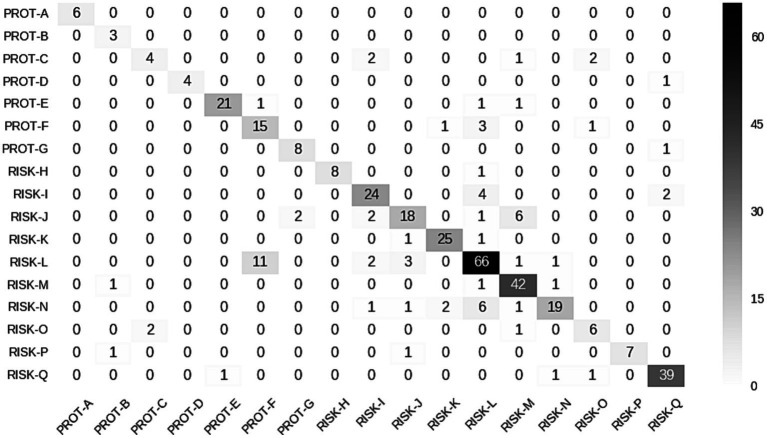
Confusion matrix showing the degree of interannotator agreement across the 17 categories.

The clear diagonal shown in the confusion matrix indicates the overall high level of agreement between the two annotators (Cohen’s kappa coefficient (κ) 0.82). There was more disagreement between Risk-I to Risk-N and Risk Q, which were the most challenging categories to define and also had the highest prevalence of words per group (Figure 4).

### N-gram frequency analysis

The majority of words used in both proximal and distal time windows are function words and pronouns. In all three representations (uni-, bi-, and tri-grams), function words (e.g., *to*, *by*, *on the*, *to the*, and *there be no*) and pronouns (e.g., *he*, *she*, *he have*, *she do*, and *that she would*) were most common.

A few verbs and nouns were also found to be informative. Reporting verbs such as say, state, and report were identified, e.g., *she say that*—*say that*—*she say she*—*she say*—*say that she*—*state that*—*state that she*—*report that she.* Other verbs included *feel* and *want*, e.g., *want to—do not want*—*not want to*—*she want to*. Nouns were only found as parts of bi- or tri-grams, e.g., *the ward*, *self-harm*—*of self-harm.*

In relation to their odds ratios, the features most informative for the distal documents were male pronouns—*his, he, he be*, while *self-harm* and female pronouns were more informative for the proximal documents. For function words such as *to and of*, the number of times they need to be present in a document for them to be distinctive is *n* = 7. The highest proximal scores are generally bi- and tri-grams, while uni- and bigrams are generally related to distal periods.

When comparing the content of the *n*-grams, many features were captured in all three representations, such as pronouns and function words. This confirmed the informativeness of unique words. The bi- and tri-grams gave a “richer picture” of why some unigrams are found informative by the classifier.

For the proximal period, the most distinctive n-grams were “self harm,” “she want to,” “of self harm,” also the distancing phrases “report that she,” “not want to.” For the distal period, “his,” “he,” “he be” and “he have” were the most informative features.

## Discussion

### Proportion of patients with documentation prior to the first suicide attempt

Our finding of approximately 40% of patients having at least one document available in their EHR prior to their first suicide attempt was congruent with a recent analysis of national trends in suicide attempts and mental health service use for adults in the US, where only approximately 40% had documented service use in the prior 12 months (24). This is of interest given they were both population-based samples but with widely different healthcare systems (25). An earlier US study conducted on an insured sample had a much higher proportion (95%) of mental healthcare contact prior to a suicide attempt (26), yet the national study by Bommersbach et al. (24) of all people who attempted suicide, regardless of insurance or treatment-seeking behaviour, paralleled our findings in the UK where we were studying the population served by the National Health Service.

Similarly, although we did not have access to primary care or Emergency Department notes, our finding of 45.0% of patients with prior records having a record documented by mental healthcare services in the past 30 days was in keeping with the frequently quoted 50% of all adults who die by suicide visiting a healthcare professional in the 4 weeks before their death (27).

### Documentation increases prior to the first suicide attempt

Our study confirmed the specific characteristics of the time period proximal to a suicide attempt which discriminates it from a distal period of lower risk. Firstly, recognition of the large increase in documentation level in the past 30 days, detectable from 60 days prior to a first suicide attempt, was only possible because of the format of recording in EHRs as opposed to paper records.

### Word categories associated with the highest and lowest risk of suicide

The category associated with the highest risk of suicide was that incorporating prescribed medications/drugs/overdose/poisoning/addiction, whereas terms associated with treatment for drug addiction or depot treatment were associated with a lower risk of suicide.

This accords with the current literature. For example, when using administrative data to predict suicide after psychiatric hospitalisation in the Veterans Health Administration System, 2 of the top 10 predictors in the Super Learner ensemble machine-learning model created were associated with drug dependence (28). Using predictive structured–unstructured interactions in EHR models, Bayramli et al. (29) showed that drug abuse or specifically named illicit drugs were the structured feature associated with greater suicide risk for many feature pairs (29).

Interestingly, this same study is one of the only published articles to specifically study apparent “protective factors” against suicide as we did (29). Concepts such as mammograms for malignant neoplasm of the breast, osteoporosis, and haemorrhoids were associated with lower risk which was analogous to our “protective” chronic physical comorbidity/symptom category. Of course, there are issues of confounding with older age, which is protective of suicide attempt risk.

Mention of a senior healthcare professional was associated with a lower risk of suicide, contrary to what was found for junior healthcare professionals. However, this is most likely confounding by indication, i.e., junior healthcare professionals being more involved in healthcare provision (30) and their roles cited in the EHR proximal to a suicide attempt, rather than being directly linked to suicide risk. Similarly, word categories which were ascribed as “protective” according to their odds ratios may simply be incidental words used to describe patients’ activities at times of low risk (e.g., food/meals/activities and items used on the ward).

A particularly interesting category was the clothing one which was associated with higher risk. Items of clothing can be used for ligatures (31), or comments can be recorded in the EHR regarding items of clothing patients bring in as property. Where a term was ambiguous, e.g., tie [which could be assigned to “implement / mechanism of self-harm or suicide attempt” (M) or “clothing” (H)], the consensus was to assign to the category conveying the highest potential risk (M). In the end, the “clothing” category was similarly categorised as of increased risk.

It was interesting that two of the categories were directly related to valence: terms with negative connotation/judgemental language being associated with increased risk, and words imparting positive connotation being “protective”. In our previous research studying six general-purpose sentiment lexicons for suicide risk assessment in EHRs (32), we found that many of the most representative keywords in the suicide-related subcorpus were not identified by any of the lexicons. The corpus word frequencies for the proximal and distal periods could be used as a guide to the inclusion of words in a novel lexicon, merging healthcare terminology as another source.

### Contextual language proximal and distal to a suicide attempt

The complementary aspect of using the n-gram method was that it allowed us to analyse word usage that captured common words/word sequences and contextual information, meaning that the proximal and distal periods could be compared based upon a contiguous sequence of n-words rather than single words (33).

Although single-word frequencies are associated with patient status and can therefore provide useful indicators of risk, single words suffer from a lack of this contextual information. For example, the same word can be used in both an affirmative and a negative context or contexts describing people other than the patient. By including surrounding context, n-grams allowed us to increase the predictive value of the textual indicators used. There is, however, some loss of sensitivity as the length of the n-grams increase: given the variable nature of language, long text sequences are less likely to provide generalisable descriptions of clinical status.

Using U.S. Veterans Administration medical records, Poulin et al. (34) generated datasets of single keywords and multi-word phrases and constructed prediction models using a machine-learning algorithm. They showed that methodologically word pairs were more useful than single words for suicide predictive model construction (34). Basic NLP features, including n-gram features, have also been used for psychiatric stressor recognition from clinical notes to study the association with suicidal behaviours (35).

Whereas gender differences in psychosocial and clinical determinants of suicide risk have been studied using EHRs (36), differences in language used have not been researched to a large extent. In our study, female pronouns being more informative for the proximal documents and male for the distal documents do not merely reflect the numbers of female and male patients, given only a slightly higher proportion of patients (55.9%) were female. Further study is needed to investigate whether clinicians document differently for female and male patients in the time leading to a suicide attempt. One study reported quoting “he/she says” is increased in records of clinician–patient interactions that involve the communication of bad news between doctor and patient (37), and this would be worth further investigation to see whether reporting styles become more formal or defensive (38) when clinicians are concerned about risk. Clinician narrative style in EHRs, e.g., use of quoted patients’ speech, has not been investigated in detail to date (39).

### Potential improvements to current EHR systems

In a review of 40 studies of the impact of EHRs on information practices in mental health contexts, Kariotis et al. (40) found that EHRs improved the amount of information documented. However, if EHRs do not include search functions or data visualisation strategies, navigating the amount of data contained in clinical notes can be challenging (40). Visualising source data from multiple domains (e.g., using Cogstack (41) or NeuroBlu (42)) can enable dynamic monitoring of risk over time, and the rate of documentation could be one aspect of this for risk of a suicide attempt. Natural language processing techniques, either rule-based, machine learning-based, or deep learning-based, can be used to extract information from clinical narratives (43). The next stage is then to build automated alerting systems with all predictive features to ensure that clinicians are notified of patients at risk so that appropriate actions can be pursued.

### Strengths and limitations

As a proxy for hospitalised suicide attempts and to study the more severe end, we purposively used HES admission data knowing that is more reliable than HES emergency department data but misses non-admitted episodes of self-harm (44, 45). Identifying suicide, self-harm, or even suicidal ideation using NLP would allow the analysis to be conducted on a broader group (46, 47).

The novel approach in this analysis was to move away from a case–control study design to consider whether it was possible to discriminate between EHR documentation proximal and distal to suicide attempts using three features of free text documentation. The main limitation was not using the features studied and other predictors in a predictive model. However, our aim was to analyse what aspects of EHR documentation and language used by clinicians change nearer to the time of a suicide attempt.

A drawback of concentrating on EHRs from a mental health trust was that we were unable to link with notes made in primary care, the general hospital, or Emergency departments as these are on separate systems.

## Conclusion

Despite its importance, clinical record keeping is often given a low priority and there is inconsistency between the entries by different healthcare professionals, yet patterns emerge in changes in documentation level, topic categories of words, and n-grams prior to a suicide attempt. More automated means of leveraging unstructured data from daily clinical practice is crucial as access to individual-level health information increases. The widespread use of EHRs has the potential to accelerate progress in developing both healthcare and research. Adopting clinical dashboards to visualise change may be particularly helpful to understand changes in suicide risk for individual patients over time.

## Data availability statement

The data analysed in this study is subject to the following licenses/restrictions: Data are owned by a third-party South London and Maudsley Biomedical Research Centre Clinical Record Interactive Search tool that provides access to anonymised data derived from electronic medical records of the South London and Maudsley National Health Service Foundation Trust. These data can only be accessed by permitted individuals from within a secure firewall (i.e., remote access is not possible, and the data cannot be sent elsewhere) in the same manner as the authors. Requests to access these datasets should be directed to rina.dutta@kcl.ac.uk.

## Ethics statement

Ethical approval/written informed consent was not required for the study of human electronic health record data in accordance with the local legislation and institutional requirements.

## Author contributions

RD was lead author and involved in conceptualising the study, methodology, analysing data, and writing and finalising the draft. GG and SV provided data curation, methodology support, and support with reviewing and editing the final draft. JD and AR provided support with methodology and analysis. RS and MH provided support with resources, and review and edit of the final draft. All authors contributed to the article and approved the submitted version.

## Footnotes

^1^ https://spacy.io
