# Supplementary material for: Identifying features of risk periods for suicide attempts using document frequency and language use in electronic health records
Source: Front Psychiatry. 2023 Dec 11;14:1217649. doi: 10.3389/fpsyt.2023.1217649 (PMC10752595; doi:10.3389/fpsyt.2023.1217649)
Supplement: Supplementary file 1 [file Table_1.DOCX]

| Group | Category description | No. of words | No. exposed documents | Examples of words used in EHR free text |
| --- | --- | --- | --- | --- |
| PROT-A | care plan | 6 | 551 | cp [care plan], cp1 |
| PROT-B | senior healthcare professional role | 3 | 116 | professor, rmo [responsible medical officer], supervisor |
| PROT-C | chronic physical comorbidity / symptom / side effect | 9 | 818 | constipation, migraine, seizure |
| PROT-D | treatment for drug addiction or depot treatment | 5 | 1,410 | methadone, buprenorphine, depot |
| PROT-E | food / meals / activities | 24 | 2,699 | rice, breakfast, crossword |
| PROT-F | positive connotation / words to describe “beginning”, “continuing” or “renewing” / any lay comment on health | 20 | 2,522 | fine, favour, commencement |
| PROT-G | place / furniture / article used on ward | 9 | 1,685 | bedspace, hallway, seat |
| RISK-H | clothing | 8 | 2,454 | coat, jacket, sock |
| RISK-I | subheadings of clerking / subsections or comments used in Mental State Examination | 30 | 6,775 | hpc [history of presenting complaint], euthymic, volume |
| RISK-J | terms / specific interventions used for a psychiatric patient, e.g. health services / management strategies / treatments / criminal justice system | 29 | 11,811 | cmht [community mental health team], hospitalisation, s136 [Section 136 Mental Health Act (1983) allows the police to take the patient to a place of safety] |
| RISK-K | time-related OR life event-related OR person / relationship | 24 | 8,048 | weekend, bereavement, nephew |
| RISK-L | suicide 'risk' related terms – words conventionally used by clinicians to describe suicide risk in notes or in the “risk assessment”  or  clinician describes patient / interaction using reassuring formal / distancing / defensive / reassuring / ‘quasi’-technical terminology | 91 | 15,523 | intention, ambivalence, planning  disclose, elaborate, reassess |
| RISK-M | implement / mechanism / site of self-harm or suicide attempt | 50 | 6,479 | ingestion, razor, scarring |
| RISK-N | negative connotations / judgemental language | 24 | 2,862 | confiscate, punish, alienation |
| RISK-O | physical symptom or sign, physical illness or treatment of a physical illness | 8 | 1,095 | coma, vomit, ulcer |
| RISK-P | junior or nursing or multidisciplinary HCP roles | 9 | 715 | assistant, cpn [community psychiatric nurse], trainee |
| RISK-Q | prescribed medications / drugs / overdose / poisoning / addiction | 41 | 6,356 | paracetamol, morphine, alcoholism |
